# Effects of Resistance Training Intervention along with Leucine-Enriched Whey Protein Supplementation on Sarcopenia and Frailty in Post-Hospitalized Older Adults: Preliminary Findings of a Randomized Controlled Trial

**DOI:** 10.3390/jcm11010097

**Published:** 2021-12-24

**Authors:** Maria Amasene, Cristina Cadenas-Sanchez, Iñaki Echeverria, Begoña Sanz, Cristina Alonso, Ignacio Tobalina, Jon Irazusta, Idoia Labayen, Ariadna Besga

**Affiliations:** 1 Department of Pharmacy and Food Science, University of the Basque Country UPV/EHU, 01006 Vitoria-Gasteiz, Spain; 2 Institute for Innovation & Sustainable Development in Food Chain (IS-FOOD), Public University of Navarra, 31006 Pamplona, Spain; cristina.cadenas@unavarra.es (C.C.-S.); idoia.labayen@unavarra.es (I.L.); 3 Department of Physiology, University of the Basque Country UPV/EHU, 48940 Leioa, Spain; inaki.echeverriag@ehu.eus (I.E.); mariabegona.sanz@ehu.eus (B.S.); jon.irazusta@ehu.eus (J.I.); 4 Biocruces Bizkaia Health Research Institute, 48903 Barakaldo, Spain; 5 Physical Rehabilitation Service, Araba University Hospital, Bioaraba Research Institute, OSI Araba, 01004 Vitoria-Gasteiz, Spain; calonbel@gmail.com; 6 Department of Nuclear Medicine, Araba University Hospital, 01004 Vitoria-Gasteiz, Spain; ignacio.tobalinalarrea@osakidetza.eus; 7 Department of Surgery Radiology and Physical Medicine, Faculty of Medicine, University of the Basque Country UPV/EHU, 01009 Vitoria-Gasteiz, Spain; 8 Department of Internal Medicine, Araba University Hospital, Bioaraba Research Institute, OSI Araba, CIBERSAM, University of the Basque Country UPV/EHU, 01004 Vitoria-Gasteiz, Spain; ariadna.besgabasterra@osakidetza.eus

**Keywords:** elderly, strength training, muscle wasting, muscle mass, myokine, myostatin, protein, leucine, whey protein, hospital

## Abstract

Resistance training and protein supplementation are expected to exert the greatest effect in counteracting muscle-wasting conditions. Myokines might play a key role, but this remains to be elucidated. The aim of this study (NCT03815201) was to examine the effects of a resistance training program with post-exercise leucine-enriched protein supplementation on sarcopenia and frailty status and on the plasma myokine concentrations of post-hospitalized older adults. A total of 41 participants were included in this 12-week resistance training intervention and randomized either to the placebo group or the protein group. Sarcopenia, frailty, body composition and blood-based myokines were measured at baseline and after 12 weeks. Both groups improved in terms of physical performance (*p* < 0.005) and frailty (*p* < 0.07) following the resistance training intervention, but without any difference between groups. Myokine concentrations did not change after the intervention in either group. Changes in myostatin concentrations were associated with greater improvements in appendicular skeletal muscle mass at the end of the intervention (*p* < 0.05). In conclusion, the implementation of resistance training programs after hospitalization in older adults should be prioritized to combat sarcopenia and frailty immediately. The results regarding myostatin should be taken as preliminary findings.

## 1. Introduction

Muscle mass loss is a widely known consequence of aging [1]. This progressive loss of muscle mass along with impaired muscle strength and function is known as sarcopenia [2]. Sarcopenia might result in physical frailty [2]. However, the cumulative decline that occurs throughout life in multiple physiological systems might also result in frailty [3]. Likewise, frailty is considered a geriatric syndrome and it might be present independently of sarcopenia [3]. Both conditions, sarcopenia and frailty, are characterized by a decline in muscle strength and poor physical function [3,4]. This can be further accelerated by physical inactivity [5].

Physical activity is proposed as one of the most effective countermeasures to address muscle-wasting conditions [5,6,7]. Specifically, resistance exercise training provides the necessary stimulus to promote muscle hypertrophy through several signaling pathways [8,9], and it is proposed that this might be further enhanced by protein supplementation in older adults [10]. Hence, leucine is considered the main precursor for activating muscle protein synthesis [11]. Likewise, it has been suggested that leucine-enriched protein supplementation might help to improve sarcopenia in older adults [12]. Muscle contraction triggers the release of myokines that influence those signaling pathways and thereby muscle growth [13].

Recent studies have examined how myokines act in response to exercise training in older adults to see whether the obtained benefits could be mediated by myokines’ responses or not. Hofman et al. concluded that the improvements observed after a resistance training program could have been mediated by the follistatin-induced blocking of the muscle degradation pathways rather than by lowering the circulating levels of myostatin [14]. Nevertheless, there is still much controversy regarding the response of both myokines to exercise, but especially myostatin, some reporting a reduction [15,16], while others show higher myostatin concentrations after an exercise intervention program [17,18]. Regarding irisin’s response to exercise training, it seems that aerobic and resistance training [19,20,21] stimulate irisin release, increasing its circulating levels in older adults, but there are also studies showing no changes [22]. The disparity shown in these studies can also be found in the studies examining the role of these myokines in muscle-wasting-related conditions [23,24,25].

There is growing interest in whether these myokines might be contributing to the muscle weakness seen with aging [26], and to see if they could serve as blood-based biomarkers [27,28]. However, there are still many aspects that need to be studied beforehand, such as myokines’ dynamics in response to exercise and aging. Therefore, the aim of this study was to examine the effects of a resistance training program along with post-exercise enriched protein supplementation on sarcopenia and frailty status as well as on plasma myokine concentrations of post-hospitalized older adults. We hypothesized that those older adults with sarcopenia and/or frailty might benefit most from resistance training along with leucine-enriched protein supplementation after hospitalization, and that those improvements might be highlighted by changes in plasma myokine concentrations. Thus, our second hypothesis was that changes in myokine plasma concentrations might predict muscle mass improvements.

## 2. Materials and Methods

### 2.1. Study Design

The Sarcopenia and Fragilidad-PROT (S&F-PROT) study is a 24-week, single-blind, randomized, placebo-controlled clinical trial (ClinicalTrials.gov ID: NCT03815201) conducted from September 2017 to July 2018 at the facilities of the Araba University Hospital in Vitoria-Gasteiz (North Spain). The study protocol (S&F-PROT) was approved by the Clinical Research Ethics Committee of the Araba University Hospital (CEIC-HUA: 2017-021) and by the Ethics Committee for Research with Biological Agents of the University of the Basque Country (CEIAB: M30/2018/201), both of which complied with the revised ethical guidelines of the Declaration of Helsinki (revision of 2013). More detailed information the main effects of the project have been published elsewhere [29] and can be found in Table S1.

Overall, the study consisted of a supervised resistance training intervention pro-gram without (placebo group) or with (protein group) post-exercise supplementation. Briefly, the protein supplement contained 20 g of whey protein isolate enriched with 3 g of leucine. Participants were randomly assigned in a parallel design (1:1 ratio, stratified by gender) to one of the two intervention groups. Randomization was conducted by the researchers involved in the intervention, so blinding was not possible for them; only the participants were blinded for allocation (intervention group). Informed consent was obtained from all the subjects involved in the study. Thereby, written informed consent was obtained from the patients to publish this paper. All of them were informed about the details of the research. The program consisted of 12 weeks of supervised training sessions followed by a period of 12 weeks of non-supervised training. However, the current study is based on the first 12 weeks of supervised training. Participants attended the training program on two non-consecutive days per week (1 h sessions). Detailed information regarding supplementation and the design of the resistance training program can be found elsewhere [29]. Briefly, the resistance training program was tailored to each participant based on 1-RM (repetition maximum) estimation, and load was then gradually increased until 70% of the estimated 1-RM was reached at the end of the intervention [29]. Sessions started with warm-up exercises followed by strengthening exercises of the upper and lower limbs and finished with 5 min of cool-down [29]. Each exercise was performed twice, and load and maximum repetition varied according to each participant [29].

### 2.2. Participants

Participants were recruited during their hospitalization at the internal medicine service or by medical recommendation at the outpatient internal medicine specialty at the Araba University Hospital [29]. The inclusion and exclusion criteria as well as the recruitment process have been described before, along with the flow diagram of participants [29]. As described before [29], the hospital recruitment process was not enough for the intervention aims, so the outpatient internal medicine service was chosen as an additional recruitment source. A total of 29 older inpatients in the hospital finally accepted to participate in the intervention program, whereas from the outpatient internal medicine service a total of 12 older adults accepted. As a result, a total of 41 older adults were randomized to either of the intervention groups. However, only 28 participants completed the intervention program (13 in the placebo group and 15 in the protein group) (Figure S1). Briefly, participants were ≥70-year-old post-hospitalized patients or geriatric outpatients with no medical contraindication nor physical and cognitive impairments for their participation in the intervention program.

### 2.3. Measurements

All measurements were performed at baseline and after 12 weeks of intervention (at week 13) by the same trained researchers.

#### 2.3.1. Sarcopenia Assessment

Sarcopenia was assessed following the proposed algorithm for finding cases by the European Working Group on Sarcopenia in Older People 2 (EWGSOP2) in the revised European consensus on the definition and diagnosis of sarcopenia [2]. For the current study, participants were first screened for muscle strength according to the handgrip strength cut-off points, and those with low muscle strength were then assessed for muscle quantity based on appendicular skeletal muscle mass (kg) cut-off points to confirm sarcopenia [2]. Handgrip strength (kg) was measured using a handled dynamometer (JAMAR ^®^ PLUS + Hand dynamometer) in a seating position, as proposed by Roberts et al. [30]. The test was performed twice, alternating each hand; the highest value was chosen and used for analysis.

#### 2.3.2. Frailty Assessment

It has been suggested that the Short Physical Performance Battery (SPPB) might help us to identify frailty in the elderly population [31,32]. The SPPB assessment methodology has been published elsewhere [33], and it consists of 3 subtests (balance, gait speed and chair stand), which contribute to the SPPB total score, which ranges from 0 to 12 [33]. The SPPB frailty threshold has been proposed at ≤9 points [31,32]. Thus, participants in the current study with a score ranging from 0 to 9 in the SPPB were classified as “frail”, whereas those scoring ≥10 were classified as “non-frail”.

#### 2.3.3. Body Composition Assessment

Body mass (kg) (OMRON HN-288, Digital Personal Scale, Kyoto, Japan) was measured barefoot following the standard protocols and height was estimated using knee height determination (SECA 220, Hamburg, Germany) [34]. For body mass index (BMI) estimation, body weight was divided by height squared (kg/m^2^). Calf circumference (cm) was measured by nonelastic tape (CESCORF, Rio Grande do Sul, Brazil) on the left side following the instructions of the Mini Nutritional Assessment questionnaire [35]. All measures were performed twice and the average was used for analysis.

Body fat, lean mass, fat-free mass and bone mass were assessed by dual-energy X-ray absorptiometry (DXA; HOLOGIC, QDR 4500, Bedford, MA, USA). Likewise, the sum of lean mass from both arms and legs was used to assess appendicular skeletal muscle mass (kg) and this was divided by height squared to assess Appendicular Skeletal Muscle Mass Index (kg/m^2^). Similarly, the Fat Mass Index was calculated as the total fat mass divided by height squared (kg/m^2^) and Fat-Free Mass Index as total fat free mass divided by height squared (kg/m^2^).

#### 2.3.4. Blood-Based Biomarkers

Biochemical parameters were obtained from fasting venous blood samples in Ethylenediaminetetraacetic acid (EDTA)-containing tubes and in serum tubes a week after the last training session. All tubes were immediately carried to the laboratory. The EDTA-containing tubes were centrifuged at 1000 g at 4 °C for 10 min, whereas serum tubes were centrifuged 90 min after blood collection at 1000 g at 20 °C for 15 min. The obtained serum aliquots from the participants were stored at −80 °C for further analysis. Myokine concentrations were quantified by commercial enzyme-linked immunosorbent assays (ELISA), following the manufacturers’ protocol. Serum myostatin (ng/mL) and follistatin (ng/mL) were measured using GDF-8/Myostatin and Follistatin Quantikine ELISA Kits, respectively (R&D Systems Inc., Minneapolis, MN, USA). Serum irisin (µg/mL) concentration was measured using an Irisin ELISA kit (AdipoGen Life Sciences, San Diego, CA, USA). For the measurement of biomarkers, the quantification was carried out by spectrophotometry with FLUOstar OPTIMA Microplate reader (ThermoFisher Scientific, Waltham, MA, USA) and Optima Control software version 2.20 (BMG, LABTECH, Ortenberg, Germany).

### 2.4. Statistical Analysis

The current study uses a secondary analysis. Indeed, sample size estimation and power analysis were calculated to determine the muscle mass increase (i.e., the primary outcome of the primary study) [29]. It was estimated that with a population size of 35 on each group, a significant alpha level of 0.05, and power > 80%, the range for a statistically detectable change in muscle mass will be 1.5–2 kg, with a standard deviation of 1.5–1.7 kg.

Data analysis was performed following the per-protocol principle. Raw scores from each variable were winsorized (when needed) to limit the influence of the outliers (i.e., extreme values). In the current study, the variables referring to myostatin baseline concentration and to changes in myostatin concentration were winsorized. The winsorization consists of replacing high/low values (percentile < 1st percentile > 99th values) with the closest (highest/lowest) valid value (1st or 99th percentile) [36]. To test our main hypothesis, changes in sarcopenia, frailty, body composition and blood-based biomarker measurements were calculated as post-intervention minus pre-intervention values (Δ = post-pre). A paired sample *t*-test was used for continuous variables, whereas a McNemar test was used for categorical variables. An analyses of covariance was carried out to examine differences in the changes in continuous variables (dependent variables) using the intervention group as the fixed factor (i.e., the placebo group and the protein group) and adjusted for baseline values.

As there were no significant differences according to muscle mass parameters nor sarcopenia and frailty statuses between both groups at the end of the intervention program, we could not test our secondary hypothesis. However, we explored the potential role of myokines in muscle mass independently of the intervention group. For this aim, linear regressions were performed between the changes in myokine concentrations (independent variable) and changes in muscle mass parameters (dependent variable) at the end of the intervention; each muscle mass parameter was adjusted for its baseline value. For the myokine concentrations that showed a significant association with any parameter of muscle mass, an additional statistical analysis was conducted. Likewise, an analysis of covariance was conducted to compare the difference in myostatin concentration between those participants that had gained vs. those that had not gained muscle mass at the end of the intervention, which was adjusted by the myostatin baseline concentration.

All statistical analyses were performed using the statistical software SPSS version 20.0 (SPSS Inc., Chicago, IL, USA) with a level of significance of α = 0.05.

## 3. Results

Table 1 shows the baseline characteristics of participants by intervention group.

### 3.1. Effects of the Intervention on Sarcopenia and Frailty Status

Although both groups improved in physical performance according to the total SPPB score (all *p* < 0.005, Table 2), only the placebo group showed a statistically significant improvement in the prevalence of frailty according to the SPPB threshold. Indeed, the number of frail participants declined from 9 to 3 after the intervention only in the placebo group (*p* < 0.05, Table 2). In the protein group, although it was not significant, five participants improved in frailty status following the intervention (*p* = 0.063, Table 2). In contrast, non-statistically significant improvements in sarcopenia status were seen for both of the intervention groups (all *p* > 0.05, Table 2). Hence, there were no statistically significant differences between groups regarding sarcopenia and frailty changes after the intervention program (all *p* > 0.05, Table 2).

### 3.2. Effects of the Intervention on Body Composition and Blood-Based Biomarkers

Table 2 shows that there were no statistically significant differences in body composition variables and myokine concentrations within each of the intervention groups nor between the two groups at the end of the intervention (all *p* > 0.05, Table 2).

### 3.3. Association of Changes in Myokine Concentration with Changes in Muscle Mass Parameters Following the Intervention Program

Table 3 shows the associations between the changes in serum concentration of each measured myokine and the respective changes in muscle mass variables. Higher in-creases in myostatin concentrations at the end of the intervention were significantly associated with greater improvements in appendicular skeletal muscle mass (kg) (β = 0.319, *p* = 0.048, Table 3). Exploratory analyses showed that those participants that gained muscle mass following the intervention program were those that had greater, but non-significant changes in myostatin concentrations (Figure S2).

## 4. Discussion

This study aimed to examine if a 12-week resistance training program along with leucine-enriched protein supplementation after each training session (two sessions/week) could be beneficial to post-hospitalized older adults (≥70 years old) in terms of improving their frailty and sarcopenia status as well as exercise-induced myokine blood concentrations. The main finding of the current study is that the addition of leucine-enriched whey protein to the resistance training program did not cause any significant improvement to frailty and sarcopenia status. Moreover, no differences between groups were observed in blood-based biomarker analyses.

The beneficial effects of resistance training on sarcopenia were not reflected in the sarcopenia status of the participants in this study. Following the EWGSOP2 algorithm, sarcopenia was confirmed based on handgrip strength (kg) and appendicular skeletal muscle mass (kg) cut-offs [2]. We previously suggested that 12 weeks of resistance training might have not been enough to see significant improvements in muscle mass measurements [29]. In this line, a meta-analysis conducted by Borde et al. concluded that 50–53 weeks were needed to observe effects on muscle mass in healthy older adults [37]. Hence, if in addition to this we considered the acute effects of hospitalization on this population [5,38,39], the time scheduled in the current study was far from optimal to observe significant improvements in muscle mass measurements. The sarcopenia criteria chosen for the current study might have had an influence on the non-significant findings, as the number of patients diagnosed with sarcopenia is lower with EWGSOP2 compared with other screening criteria [40,41]. Overall, it might be due to these factors along with the multifactorial nature of sarcopenia [42] and the small sample size that the current study failed to observe any beneficial effects of resistance training on sarcopenia status. Hence, the number of those diagnosed with this condition was small. In contrast, according to frailty status, more than half of the older adults in the placebo group and 40% in the protein group were frail at baseline. Performance within the SPPB was significantly improved in both intervention groups regardless of protein-enriched supplementation. This improvement in performance did not represent a significant improvement in the frailty status of older adults in the protein group, but it did in the placebo group. Nevertheless, it is worth mentioning that, although non-significant, five of those frail older adults at baseline in the protein group were not frail after the intervention program. Again, the small sample size might have hampered our ability to observe a significant effect of resistance training on frailty status in this intervention group. Their performance in the SPPB improved significantly, so it might be suggested that with a greater sample size their frailty status would have been improved accordingly or that, as suggested for sarcopenia, more time (≥12 weeks) is needed to observe the beneficial effects in frailty status. Nevertheless, these results highlight the efficacy of resistance training programs on improving the physical performances of older adults, and thereby preventing the onset or progression of frailty in its early stages [43].

In the current study, the concentrations of myostatin, follistatin and irisin did not significantly change after the intervention program in either of the groups or between both groups (placebo group vs. protein group). The results published by Hofmann et al. are not in line with the results we observed for follistatin, but they are in accordance with those observed for myostatin levels showing no significant changes after the intervention [14]. However, other authors have reported significant decreases [15,16] or increases [17,18] in serum myostatin levels. According to irisin, our results are in line with Hecksteden et al., showing no effects of the intervention program on its serum levels [22].

Follistatin and irisin, both known for their anabolic effects [13], did not show any association with either of the measured muscle mass parameters in the current study. Surprisingly, myostatin showed a significant association with appendicular skeletal muscle mass (kg), suggesting that the change in myostatin concentrations (post–pre values of myostatin concentrations) might predict or reflect the change in appendicular skeletal muscle mass (kg) (Δ ASMM (kg)) following the intervention program. Indeed, we also observed that those older adults that increased, although non-significantly, in appendicular skeletal muscle mass (kg) and Fat-Free Mass Index (kg/m^2^) after the intervention program were those that had the greatest change in myostatin concentration. Arrieta et al. did not observe a significant association between the increase in lean mass and myostatin concentrations following a multicomponent physical exercise intervention [18]. Despite this, they observed that improvements in physical parameters after the intervention were positively associated with higher myostatin levels in men [18]. In addition, in the Vienna Active Ageing Study, it was observed that lower levels of myostatin at baseline were associated with a smaller increase in muscle mass or even muscle mass loss [14]. These results might have two possible explanations. On the one hand, as proposed by other authors [18,44], it could be that in light of the anabolic stimulus in the resistance training program, myostatin serum levels might have increased to restrain unlimited muscle mass growth, acting as a chalone. On the other hand, as has been suggested before, myostatin might be required for myogenesis to take place, despite being a negative regulator of it [45]. Thus, in line with our results, it could be that myostatin might have some implication in muscle mass gains, albeit following a resistance training program in older adults. In contrast, one could also speculate that simply the effects at the cellular level might not correspond with a direct decrease in the serum levels of myostatin, albeit in the short term [46]. For example, it could be that myostatin gene expression is suppressed and/or that its actions, such as decreasing mTORC1 signaling pathways [47], are blocked due to the stimuli of resistance training favoring muscle mass growth. However, the expected decrease in myostatin levels might take a longer time to occur. Thus, just measuring serum myostatin levels might lead to misleading conclusions. Nevertheless, these are just speculations as the sample size in the current study was small and does not permit us to reach conclusive statements.

### Strength and Limitations

Although our results regarding myostatin’s role suggest a research line to follow, we should not omit the small sample size as a limitation of the current study. There are still many aspects that need to be clarified, such as the sex interaction. Some authors have suggested that myostatin might have a homeostatic role in males while in females might contribute to the age-related muscle mass loss [25]. However, how these opposed roles of myostatin affect the response to an anabolic stimulus within each gender needs to be established. The time-point chosen in this study for the collection of blood samples (1 week after finishing the training program) should be considered as a limitation too. Although, the time-point at which the serum myokine concentration might represent exercise-induced myokine released by muscle is not well established; we could have determined this if we had measured them between 24 and 72 h after the last training session. Nevertheless, a recent meta-analysis showed that myostatin gene expression was downregulated in the long term within the skeletal muscle [48]. So, it might be speculated that the increase observed in myostatin serum concentration in this study was due to the acute response of muscle mass to resistance training. However, this is just speculation and this as well as the time course of other myokines should be studied in future studies. Thus, future studies, regarding these issues and others, with larger sample sizes are needed before general conclusions are made. The small sample size in this study was also stated as an important limitation explaining the lack of significant improvements in muscle mass after the intervention [29] along with the inability of DXA to detect differences smaller than 1.0 kg [49]. These limitations might have also not allows us observe significant improvements in the sarcopenia status of participants. The single-blinded nature of the study should also be taken as a limitation. One of the strengths of the current study is that, to our knowledge, this is the first randomized controlled trial including older adults immediately after hospitalization in a resistance training program with post-exercise leucine-enriched protein supplementation.

## 5. Conclusions

This study reinforces resistance training as a primary countermeasure to combat and/or prevent frailty in post-hospitalized older adults. Although significant improvements in frailty status were only observed in one intervention group, older adults in both groups significantly improved in physical performance regardless of the protein supplementation. We consider this an important point to highlight, as with 12 weeks of resistance training, we started to observe beneficial effects on physical performance in older post-hospitalized adults. Likewise, we suggest that, if prolonged, sarcopenia and frailty status will improve accordingly. Future studies should be conducted to establish the minimum length needed to observe significant improvements in the sarcopenia and frailty status of post-hospitalized older adults. Studies examining the effective dose, frequency and meal distribution of protein supplementation are also needed in these muscle-wasting conditions. Hence, due to their multifactorial nature, it might be that protein supplementation should be accompanied by other nutritional supplements [50].

In addition, our results regarding myostatin’s role should be taken as preliminary evidence that needs to be proven in future studies with larger sample sizes. Future studies with larger sample sizes need to be conducted to understand how myostatin responds to training stimuli at the cellular as well as systemic level and if these responses correspond with the training outcomes observed in different contexts.

## Figures and Tables

**Table 1:** Characteristics of participants in the study at baseline.

	N	Total	N	Placebo Group	N	Protein Group
Age (years)	41	82.1 (5.89)	20	81.2 (6.14)	21	82.9 (5.67)
Women (N, %)	41	22, 53.7	20	10, 50	21	12, 57.1
Body mass (kg)	40	72.4 (15.6)	19	77.5 (17.02)	21	67.8 (12.92)
Height (m)	40	1.6 (0.1)	19	1.6 (0.1)	21	1.6 (0.1)
BMI (Kg/m^2^)	40	29.1 (5.22)	19	31.1 (5.83)	21	27.4 (3.95)
Fat mass index (kg/m^2^)	40	10.1 (3.63)	19	11.4 (3.81)	21	8.9 (3.12)
Fat Free mass index (kg/m^2^)	40	18.4 (2.46)	19	19.0 (3.09)	21	17.8 (1.56)
Calf circumference (cm)	40	35.6 (4.41)	19	36.3 (4.94)	21	34.9 (3.88)
Sarcopenic assessment						
Handgrip strength (kg)	41	25.3 (7.63)	20	24.5 (7.16)	21	26.1 (8.14)
Appendicular skeletal muscle mass (kg)	41	18.1 (3.98)	20	18.7 (4.55)	21	17.5 (3.35)
Appendicular Skeletal Muscle Mass Index (kg/m^2^)	40	7.2 (1.15)	19	7.5 (1.41)	21	7.1 (0.83)
Sarcopenic (N, %)	41	6, 14.6	20	3, 15.0	21	3, 14.3
Frailty assessment						
SPPB total score	41	9.1 (2.40)	20	8.7 (2.43)	21	9.5 (2.36)
Frail (N, %)	41	21, 51.2	20	13, 65.0	21	8, 38.1
Blood based biomarkers						
Myostatin (ng/mL)	40	3.3 (2.03)	20	3.5 (2.39)	20	3.1 (1.64)
Follistatin (ng/mL)	40	2.9 (1.26)	20	3.0 (1.46)	20	2.8 (1.06)
Follistatin to myostatin ratio	40	1.2 (0.95)	20	1.3 (1.08)	20	1.2 (0.82)
Irisin (µg/mL)	30	8.2 (3.96)	14	9.3 (4.17)	16	7.4 (3.66)

Data are presented as mean (standard deviation) unless other is indicated. BMI: body mass index. SPPB: Short Physical Performance Battery.

**Table 2:** Body composition, nutritional status and physical function in elderly patients before (pre) and after (post) their participation in the resistance exercise intervention program plus protein supplementation (Protein group) or placebo (placebo group) (analyses *per protocol*).

	Placebo Group				Protein Group				Differences between Groups		
	N	Pre	Post	*p* *	N	Pre	Post	*p* *	Δ Placebo	Δ Protein	*p* ^†^
Sarcopenic assessment											
Handgrip strength (kg) ^§^	13	24.8 (7.63)	24.5 (7.32)	0.704	15	26.9 (6.85)	26.6 (6.50)	0.699	−0.2 (2.28)	−0.4 (3.66)	0.883
Appendicular skeletal muscle mass (kg)	13	18.3 (4.65)	18.5 (3.60)	0.681	15	17.3 (2.81)	17.3 (2.78)	0.787	0.2 (1.64)	−0.0 (0.77)	0.282
Appendicular Skeletal Muscle Mass Index (kg/m^2^)	13	7.4 (1.50)	7.5 (1.16)	0.561	15	6.9 (0.64)	6.9 (0.66)	0.794	0.1 (0.63)	−0.0 (0.30)	0.150
Sarcopenic (N, %)	13	2, 15.4	2, 15.4	1.000	15	3, 20.0	2, 13.3	1.000	0.0, 0.0	−1, 6.7	1.000
Frailty assessment											
SPPB score total ^§^	13	8.7 (2.36)	10.3 (1.89)	**0.001**	15	10.1 (1.58)	11.3 (0.96)	**0.002**	1.6 (1.39)	1.2 (1.21)	0.634
Frail (N, %)	13	9, 69.2	3, 23.1	**0.031**	15	6, 40.0	1, 6.7	0.063	−6, 46.2	−5, 33.3	0.700
Body composition											
Fat mass index (kg/m^2^)	13	11.1 (4.35)	11.1 (4.67)	0.843	15	9.0 (3.02)	9.1 (2.81)	0.418	−0.0 (0.64)	0.1 (0.55)	0.460
Fat Free mass index (kg/m^2^)	13	19.0 (3.29)	18.9 (2.80)	0.524	15	17.8 (1.37)	17.9 (1.45)	0.375	−0.2 (0.94)	0.1 (0.40)	0.731
Calf circumference (cm)	13	36.1 (5.28)	36.2 (5.29)	0.545	15	35.2 (3.64)	35.5 (3.44)	0.138	0.1 (0.82)	0.3 (0.81)	0.621
Blood based biomarkers											
Myostatin (ng/mL)	13	3.5 (2.8)	3.1 (1.85)	0.444	15	3.0 (1.85)	2.9 (1.45)	0.938	−0.3 (1.55)	−0.0 (1.29)	0.799
Follistatin (ng/mL)	13	3.1 (1.26)	3.3 (1.73)	0.482	15	2.8 (1.08)	2.9 (1.49)	0.447	0.3 (1.36)	0.2 (0.87)	0.816
Follistatin to myostatin ratio	13	1.4 (1.09)	1.9 (2.29)	0.381	15	1.3 (0.91)	1.5 (1.58)	0.370	0.4 (1.79)	0.2 (0.90)	0.720
Irisin (µg/mL)	13	9.3 (4.3)	7.9 (2.99)	0.161	15	7.5 (3.76)	7.7 (3.57)	0.814	−1.4 (3.31)	0.2 (3.85)	0.624

Data are presented as mean (standard deviation). ^§^ Data from Amasene et al. (2019) [29]. * *p* indicates statistical differences between pre and post values by paired *t*-Student test (continuous variables) and McNemar test (categorical variables). Δ placebo indicates the difference between pre and post values in the placebo group (Δ = post-pre); Δ protein indicates the difference between pre and post values in the protein group (Δ = post-pre). †*p* indicates statistical significance between Δ placebo and Δ protein, analyzed by analysis of covariance (continuous variables) or chi square test (categorical variables), adjusted for baselines values.

**Table 3:** Associations of changes in myokines with changes in muscle mass parameters after the intervention.

	Δ Handgrip Strength (kg)		Δ ASMM (kg)		Δ ASMMI (kg/m^2^)		Δ FFMI (kg/m^2^)	
	β	*p*	β	*p*	β	*p*	β	*p*
Δ Myostatin (ng/mL)	0.043	0.819	0.319	0.048	0.256	0.128	0.243	0.165
Δ Follistatin (ng/mL)	−0.014	0.941	0.097	0.561	0.061	0.716	0.238	0.157
Δ Follistatin to Myostatin ratio	−0.055	0.771	0.066	0.693	0.049	0.771	0.128	0.455
Δ Irisin (µg/mL)	0.101	0.590	−0.084	0.615	−0.075	0.659	−0.130	0.445

β: standardized beta coefficient. ASMM: appendicular skeletal muscle mass; ASMMI: Appendicular Skeletal Muscle Mass Index; FFMI: Fat-Free Mass Index. Linear regression tests adjusted for baseline values of each muscle mass parameter.

## Supplementary Materials

The following are available online at https://www.mdpi.com/article/10.3390/jcm11010097/s1, Figure S1: Flow diagram of participants, Figure S2: Comparison of the difference in myostatin concentration between participants with muscle mass gains vs. no muscle mass gains after the intervention program. Analysis of covariance adjusted for myostatin baseline concentration. Table S1: CONSORT-SPI Checklist.
