# Supplementary material for: Effects of Resistance Training Intervention along with Leucine-Enriched Whey Protein Supplementation on Sarcopenia and Frailty in Post-Hospitalized Older Adults: Preliminary Findings of a Randomized Controlled Trial
Source: J Clin Med. 2021 Dec 24;11(1):97. doi: 10.3390/jcm11010097 (PMC8745511; doi:10.3390/jcm11010097)
Supplement: Supplementary file 1 [file jcm-11-00097-s001.zip › Supplementary files/Figure S2.pdf]

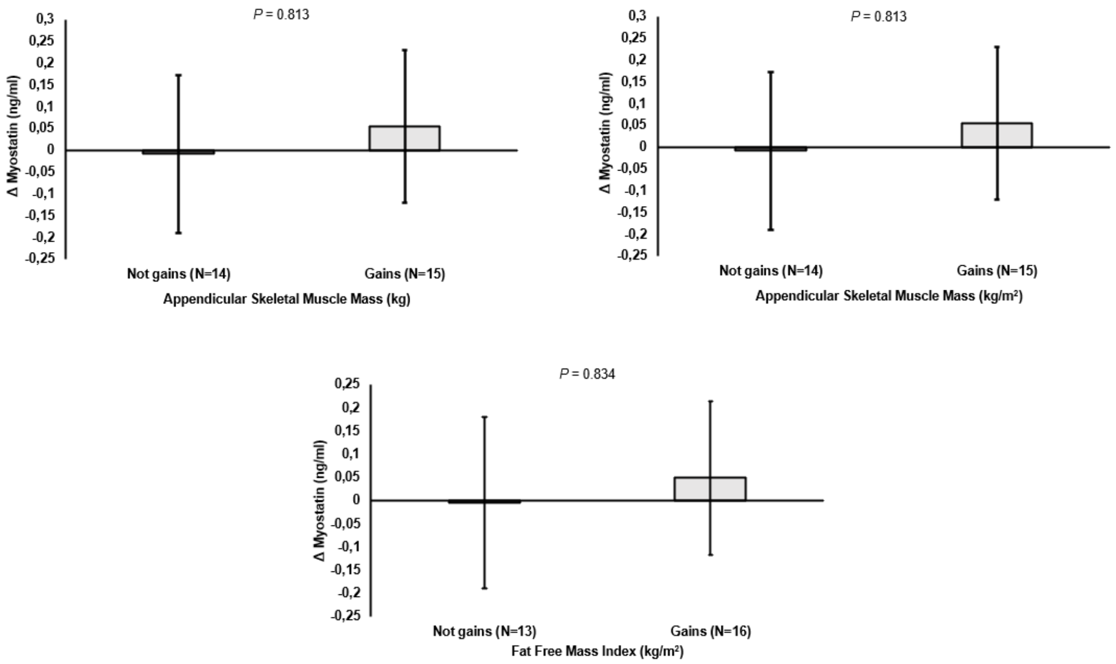

Figure S2. Comparison of the difference in myostatin concentration between participants with muscle mass gains vs. not muscle mass gains after the intervention program. Analysis of covariance adjusted for myostatin baseline concentration.
