# Supplementary material for: Effects of Resistance Training Intervention along with Leucine-Enriched Whey Protein Supplementation on Sarcopenia and Frailty in Post-Hospitalized Older Adults: Preliminary Findings of a Randomized Controlled Trial
Source: J Clin Med. 2021 Dec 24;11(1):97. doi: 10.3390/jcm11010097 (PMC8745511; doi:10.3390/jcm11010097)
Supplement: Supplementary file 1 [file jcm-11-00097-s001.zip › Supplementary files/Table S1_CONSORT-SPI Checklist_JCM.pdf]

CONSORT-SPI 2018 Checklist

SECTION	ITEM #	CONSORT-SPI 2010	CONSORT-SPI 2018	REPORTED ON PAGE #
**TITLE AND ABSTRACT**				
	1a	Identification as a randomised trial in the title		Page 1
	1b	Structured summary of trial design, methods, results, and conclusions (for specific guidance see CONSORT for Abstracts)	Refer to CONSORT extension for social and psychological intervention trial abstracts	Page 1, abstract
**INTRODUCTION**				
Background and Objectives	2a	Scientific background and explanation of rationale		Page 1-2, line 46-82
	2b	Specific objectives or hypotheses	If pre-specified, how the intervention was hypothesied to work	Page 2, line 79-87
**METHODS**				
Trial Design	3a	Describe of trial design (such as parallel, factorial), including allocation ratio	If the unit of random assignment is not the individual, please refer to CONSORT for Cluster Randomized Trials	Page 2-3, line 88-96.<br>Page 3, line 99-105
	3b	Important changes to methods after trial commencement (such as eligibility criteria), with reasons		No changes
Participants	4a	Eligibility criteria for participants	When applicable, eligibility criteria for settings and those delivering the interventions	Page 3, line 122-123, line 131-133
	4b	Settings and locations where the data were collected		Page 3, line 120-122
Interventions	5	The interventions for each group with sufficient details to allow replication, including how and when they are actually administered		Page 3, line 108-118
	5a		Extent to which interventions were actually delivered by providers and taken up by participants as planned	

	5b		Where other informational materials about delivering the intervention can be accessed	Amasene et al. (2019) (doi:10.3390/nu11102337)
	5c		When applicable, how intervention providers were assigned to each group	Not applicable
Outcomes	6a	Completely defined pre-specified outcomes, including how and when they were assessed		Page 3-4, line 135-185
	6b	Any changes to trial outcomes after the trial commenced, with reasons		No changes
Sample Size	7a	How sample size was determined		Page 4, line 187-191
	7b	When applicable, explanation of any interim analyses and stopping guidelines		Not applicable
**RANDOMISATION**				
Sequence generation	8a	Method used to generate the random allocation sequence		Page 3, line 102-105
	8b	Type of randomisation; detail of any restriction (such as blocking and block size)		Page 3, line 102-105
Allocation concealment mechanism	9	Mechanism used to implement the random allocation sequence, describing any steps taken to conceal the sequence until interventions were assigned		Page 3, line 102-105
Implementation	10	Who generated the random allocation sequence, who enrolled participants, and who assigned participants to interventions§		Page 3, 103-105
Awareness of assignment	11a	Who was aware of intervention assignment after allocation (for example, participants, providers, those assessing outcomes), and how any masking was done		Page 3, 103-105

	11b	If relevant, description of the similarity of interventions		
Analytical methods	12a	Statistical methods used to compare group outcomes	How missing data were handled, with details of any imputation method	Page 4-5, line 192-215
	12b	Methods for additional analyses, such as subgroup analyses, adjusted analyses, and process evaluations		Page 4-5, line 192-215
**RESULTS**				
Participant flow (a diagram is strongly recommended)	13a	For each group, the numbers randomly assigned, receiving the intended intervention, and analysed for the outcomes	Where possible, the number approached, screened, and eligible prior to random assignment, with reasons for non-enrolment	Figure S1. (Flow-diagram)
	13b	For each group, losses and exclusions after randomisation, together with reasons		Figure S1. (Flow-diagram)
Recruitment	14a	Dates defining the periods of recruitment and follow-up		Page 2, line 92
	14b	Why the trial ended or was stopped		
Baseline data	15	A table showing baseline characteristics for each group	Include socioeconomic variables where applicable	Page 5
Numbers analysed	16	For each group, number included in each analysis and whether the analysis was by original assigned groups		Page 7
Outcomes and estimation	17a	For each outcome, results for each group, and the estimated effect size and its precision (such as 95% confidence interval)	Indicate availability of trial data	Page 5-8, line 219-252
	17b	For binary outcomes, the presentation of both absolute and relative effect sizes is recommended		
Ancillary analyses	18	Results of any other analyses performed, including subgroup analyses, adjusted analyses, and process evaluations, distinguishing pre-specified from exploratory		Page 5, 204-215.<br>Page 8 (Table 3)<br>Figure S2.

Harms	19	All important harms or unintended effects in each group (for specific guidance see CONSORT for Harms)		
**DISCUSSION**				
Limitations	20	Summarize the main results (including an overview of concepts, themes, and types of evidence available), link to the review questions and objectives, and consider the relevance to key groups.	Trial limitations, addressing sources of potential bias, imprecision, and, if relevant, multiplicity of analyses	Page 8-9, line 258-335<br>Page 9-10, line 337-362
Generalisability	21	Discuss the limitations of the scoping review process.	Generalisability (external validity, applicability) of the trial findings	Page 9-10, line 337-362
Interpretation	22	Provide a general interpretation of the results with respect to the review questions and objectives, as well as potential implications and/or next steps.	Interpretation consistent with results, balancing benefits and harms, and considering other relevant evidence	Page 10, line 364-381
**IMPORTANT INFORMATION**				
Registration	23	Registration number and name of trial registry		Page 2, line 90-91
Protocol	24	Where the full trial protocol can be accessed, if available		
Declaration of Interests	25	Sources of funding and other support; role of funders	Declaration of any other potential interests	Page 11, line 396-400<br>Page 11, line 413-415
Stakeholder investments	26a		Any involvement of the intervention developer in the design, conduct, analysis, or reporting of the trial	Page 10, line 387-395
	26b		Other stakeholder involvement in trial design, conduct, or analyses	No
	26c		Incentives offered as part of the trial	No incentives offered

This table lists items from the CONSORT 2010 checklist (with some modifications for social and psychological intervention trials) and additional items in the CONSORT-SPI 2018 extension. Empty rows in the ‘CONSORT-SPI 2018’ column indicate that there is no extension to the CONSORT 2010 item

*We strongly recommended that the CONSORT-SPI 2018 Explanation and Elaboration (E&E) document be reviewed when using the CONSORT-SPI 2018 checklist for important clarifications on each item

§An extension item for cluster trials exists for this CONSORT 2010 item

Citations

Montgomery, P., Grant, S., Mayo-Wilson, E., Macdonald, G., Michie, S., Hopewell, S., & Moher, D. (2018). Reporting randomised trials of social and psychological interventions: the CONSORT-SPI 2018 Extension. *Trials*, *19*(1), 407.

Grant, S., Mayo-Wilson, E., Montgomery, P., Macdonald, G., Michie, S., Hopewell, S., & Moher, D. (2018). CONSORT-SPI 2018 Explanation and Elaboration: guidance for reporting social and psychological intervention trials. *Trials*, *19*(1), 406.
