# Supplementary figures and images for: Effects of Resistance Training Intervention along with Leucine-Enriched Whey Protein Supplementation on Sarcopenia and Frailty in Post-Hospitalized Older Adults: Preliminary Findings of a Randomized Controlled Trial
Source: J Clin Med. 2021 Dec 24;11(1):97. doi: 10.3390/jcm11010097 (PMC8745511; doi:10.3390/jcm11010097)

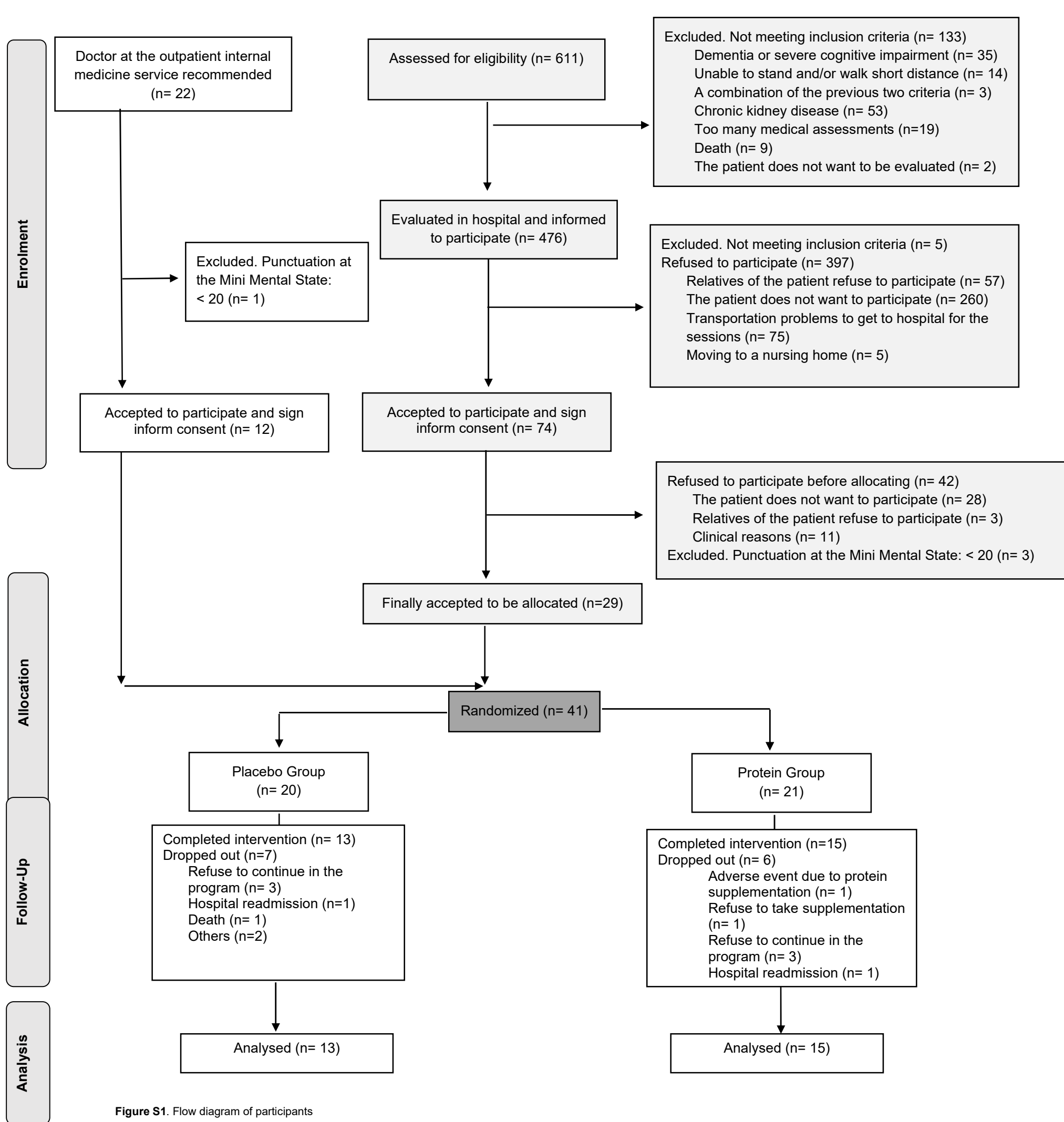

**Figure S1.** Flow diagram of participants

Supplement: Supplementary file 1 [file jcm-11-00097-s001.zip › Supplementary files/Figure S1_flow-diagram.pdf]
